# Non-Adrenergic Vasopressors in Patients with or at Risk for Vasodilatory Shock. A Systematic Review and Meta-Analysis of Randomized Trials

**DOI:** 10.1371/journal.pone.0142605

**Published:** 2015-11-11

**Authors:** Alessandro Belletti, Mario Musu, Simona Silvetti, Omar Saleh, Laura Pasin, Fabrizio Monaco, Ludhmila A. Hajjar, Evgeny Fominskiy, Gabriele Finco, Alberto Zangrillo, Giovanni Landoni

**Affiliations:** 1 Department of Anaesthesia and Intensive Care, IRCCS San Raffaele Scientific Institute, Milan, Italy; 2 Department of Medical Sciences “M. Aresu”, Cagliari University, Cagliari, Italy; 3 Surgical Intensive Care Unit, Department of Cardiopneumology, University of São Paulo, São Paulo, Brazil; 4 Department of Anaesthesiology and Intensive Care, Academician EN Meshalkin Novosibirsk State Budget Research Institute of Circulation Pathology, Novosibirsk, Russia; 5 Vita-Salute San Raffaele University, Milan, Italy; Bambino Gesù Children's Hospital, ITALY

## Abstract

**Introduction:**

Hypotensive state is frequently observed in several critical conditions. If an adequate mean arterial pressure is not promptly restored, insufficient tissue perfusion and organ dysfunction may develop. Fluids and catecholamines are the cornerstone of critical hypotensive states management. Catecholamines side effects such as increased myocardial oxygen consumption and development of arrhythmias are well known. Thus, in recent years, interest in catecholamine-sparing agents such as vasopressin, terlipressin and methylene blue has increased; however, few randomized trials, mostly with small sample sizes, have been performed. We therefore conducted a meta-analysis of randomized trials to investigate the effect of non-catecholaminergic vasopressors on mortality.

**Methods:**

PubMed, BioMed Central and Embase were searched (update December 31^st^, 2014) by two independent investigators. Inclusion criteria were: random allocation to treatment, at least one group receiving a non-catecholaminergic vasopressor, patients with or at risk for vasodilatory shock. Exclusion criteria were: crossover studies, pediatric population, non-human studies, studies published as abstract only, lack of data on mortality. Studied drugs were vasopressin, terlipressin and methylene blue. Primary endpoint was mortality at the longest follow-up available.

**Results:**

A total of 1,608 patients from 20 studies were included in our analysis. The studied settings were sepsis (10/20 studies [50%]), cardiac surgery (7/20 [35%]), vasodilatory shock due to any cause (2/20 [19%]), and acute traumatic injury (1/20 [5%]). Overall, pooled estimates showed that treatment with non-catecholaminergic agents improves survival (278/810 [34.3%] versus 309/798 [38.7%], risk ratio = 0.88, 95% confidence interval = 0.79 to 0.98, p = 0.02). None of the drugs was associated with significant reduction in mortality when analyzed independently. Results were not confirmed when analyzing studies with a low risk of bias.

**Conclusions:**

Catecholamine-sparing agents in patients with or at risk for vasodilatory shock may improve survival. Further researches on this topic are needed to confirm the finding.

## Introduction

Severe hypotension is common among critically ill patients. When mean arterial pressure (MAP) falls below a critical threshold, inadequate tissue perfusion ensues, leading to multiple organ dysfunction and death [1–2]. Therefore, prompt treatment of hypotension is mandatory in critically ill patients [2–4].

Fluid administration is often the first-line therapy. However, fluid resuscitation alone is often insufficient to restore an adequate perfusion pressure, and administration of vasopressors becomes necessary. Catecholamines and in particular norepinephrine are the most frequently used vasopressor agents [5]; however, catecholamines have well-known side effects such as increased myocardial oxygen consumption and arrhythmias that may ultimately worsen patients’ prognosis despite positive hemodynamic effects [6,7].

Moreover, late-phase shock often become unresponsive to treatment with catecholamines, due to several mechanisms including desensitization of adrenergic receptors, alteration in nitric oxide (NO) production pathway, and opening of ATP-sensitive K^+^ channels [8,9].

Therefore, in recent years, catecholamine-sparing agents have emerged as promising alternative drugs for treatment of shock [10–13]. Currently, the most frequently used catecholamine-sparing vasopressor agents are vasopressin, its long half-life derivative, terlipressin, and methylene blue [13,14].

Several observational studies and case-report of use of these agents in different forms of shock can be found in literature; however, only few randomized controlled trials (RCTs) have been published, mostly with small sample size and with hemodynamic rather than clinical endpoints.

We therefore conducted a meta-analysis of RCTs to investigate the effect of vasopressin, terlipressin, and methylene blue on mortality in patients with or at risk for vasodilatory shock.

## Methods

### Search Strategy

Pertinent studies were independently searched in PubMed, Embase, BioMedCentral and the Cochrane Central Register of clinical trials (updated December 31^st^ 2014) by two investigators. Our search strategy aimed to include any RCTs ever performed in humans with non-catecholaminergic vasopressors in any clinical setting. In addition, we employed backward snowballing (i.e., scanning of references of retrieved articles and pertinent reviews) to obtain further studies. No language restriction was employed. The search strategy for PubMed [15] is available as Supplementary Material.

### Study Selection

References were first independently examined at a title/abstract level by two investigators, with divergences resolved by consensus, and then, if potentially pertinent, retrieved as complete articles. Inclusion criteria for potentially relevant studies were random allocation to treatment, at least one group receiving vasopressin, terlipressin, or methylene blue [13,14], patients with or at risk for vasodilatory shock. Established vasodilatory shock was considered as per author definition, regardless of the cause. We considered at risk for vasodilatory shock patients with sepsis, patients undergoing cardiac surgery, and patients at risk for developing shock due to other acute, severe medical conditions (i.e. major hemorrhage) [9,11,13]. Patients considered “at risk” received the study drug before development of shock to prevent hemodynamic derangement and consequent low tissue perfusion and organ failure. The exclusion criteria were overlapping populations (in this case we referred to the first article published while retrieved data from the article with the longest follow-up available), non-adult patients (age < 16), animal studies, studies published as abstract only and lack of data on mortality. Studies on NO synthase inhibitors other than methylene blue (i.e. tilarginine acetate) were excluded as these agents are not currently in use. Two investigators independently assessed adherence to selection criteria and selected studies for the final analysis, with divergences resolved by consensus.

### Data Abstraction and Study

Baseline, procedural, outcome and follow-up data were independently abstracted by two investigators. Corresponding authors of retrieved articles reporting no data on mortality were contacted by email to obtain missing data. If a trial reported multiple comparisons, the non-study drugs comparators were aggregated as a single control group. The primary endpoint of our meta-analysis was mortality at the longest follow-up available.

### Internal Validity and Risk of Bias Assessment

The internal validity and risk of bias of included trials was appraised by two independent investigators according to the latest version of the “Risk of bias assessment tool” developed by The Cochrane collaboration [16], with divergences resolved by consensus. To assess the presence of publication bias, Egger’s linear regression test and Begg’s adjusted-rank correlation test were performed.

### Data Analysis and Synthesis

Computations were performed with RevMan (Review Manager version 5.3, The Nordic Cochrane Center, The Cochrane Collaboration, Copenaghen, 2014) and Stata (Stata Statistical Software: Release 13, StataCorp LP, College Station, Texas). Hypothesis of statistical heterogeneity was tested by means of Cochran Q test, with statistical significance set at the two-tailed 0.10 level, whereas extent of statistical consistency was measured with I^2^, defined as 100% X (Q-df)/Q, where Q is Cochran’s heterogeneity statistic and df the degrees of freedom. Mortality data were extrapolated to compute the individual and pooled risk ratio (RR) with pertinent 95% confidence interval (CI), by means of inverse variance method and with a random-effect model. In addition to the principal analysis considering all the studies that fulfilled inclusion criteria, we also performed secondary analyses to investigate specific clinical settings and the effect of the different drugs, and we analysed the studies reporting 28/30-day mortality. Sensitivity analyses were performed by sequentially removing each study and reanalysing the remaining dataset (producing a new analysis for each study removed) and by analysing only data from studies with low risk of bias. Statistical significance was set at the two-tailed 0.05 level for hypothesis testing. Unadjusted p values are reported throughout. All data were analysed according to the intention-to-treat principle. This study was performed in compliance with The Cochrane Collaboration and Preferred Reporting Items for Systematic Reviews and Meta-Analyses guidelines [17–20] (S1 Appendix).

## Results

### Study Characteristics

**Fig 1:**
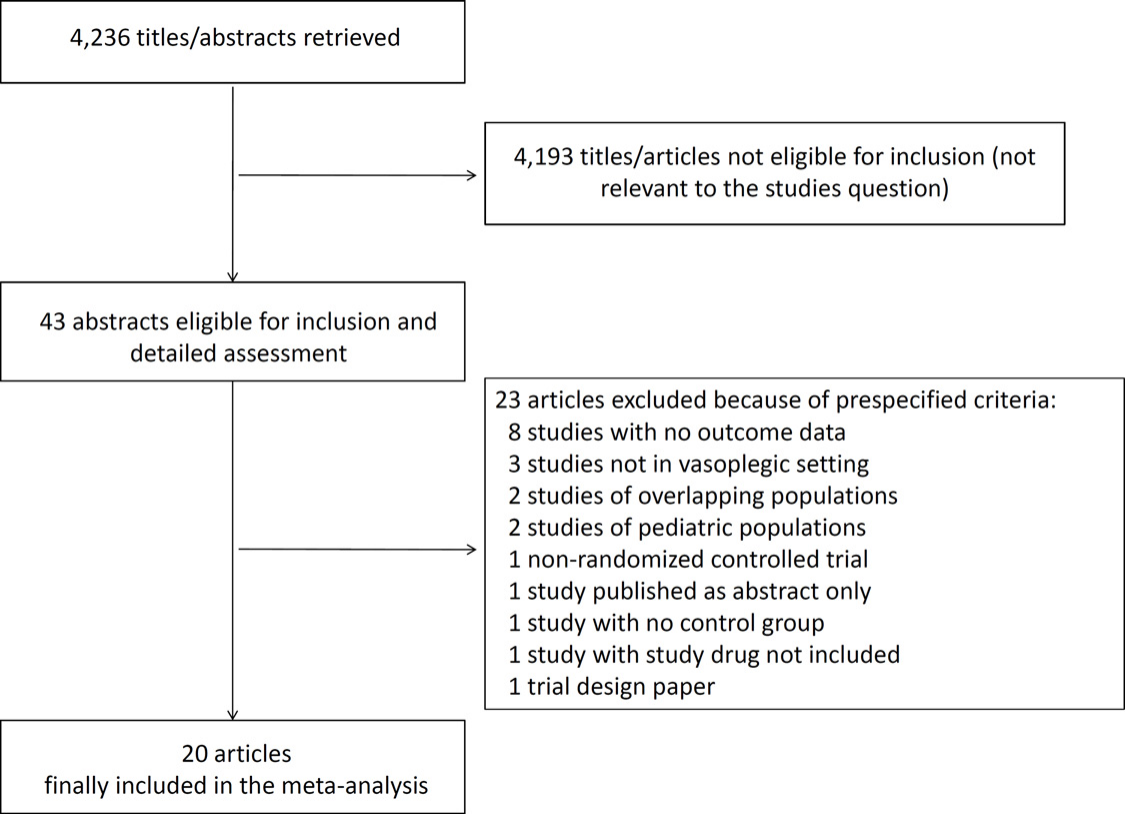
Flow diagram for selection of articles.

Database searches and snowballing yielded a total of 4,236 articles. After exclusion of 4,193 nonpertinent titles or abstracts, 43 papers were retrieved in complete form and assessed according to selection criteria (Fig 1). A total of 23 papers were excluded due to pre-specified selection criteria, leaving 20 manuscripts [10,21–39] for the final analysis. The flow chart to select the final 20 manuscripts is detailed in Fig 1.

A complete list of excluded studies, together with reason for exclusion, is provided as supplementary material (Table A in S2 Appendix).

The 20 included manuscripts randomized 1,608 patients (810 to study drugs and 798 to control) (Table 1). A total of 10 studies (50%) were performed in the setting of sepsis [10,21,25–27,30,32,34,35,39], 7 (35%) in the setting of cardiac surgery [24,28,31,33,36–38], two (10%) in the setting of vasodilatory shock due to any cause [23,29], and one (5%) in the setting of acute traumatic injury [22]. Different comparators were used: placebo in 10 study arms [22,24,26,28,30–33,36,38], standard treatment in five arms [23,29,34,37,39], norepinephrine in four arms [10,21,27,35], and dopamine in one arm [25]. One study reported multiple comparisons: patients were randomized to receive vasopressin or terlipressin or norepinephrine [35]. Duration of follow-up varied among the different studies: five studies followed-up patients until discharge from intensive care unit (ICU) [23,27,29,34,35], seven until hospital discharge [21,24,28,32,33,37,38], six until 28/30 days [10,22,25,26,36,39] and two until 90 days [10,39]. A total of 616 patients were randomized to vasopressin, 76 to terlipressin, and 118 to methylene blue. Three studies were multicentric [10,27,28]. In five studies, the study drug was administered prophylactically to prevent the onset of severe hypotension and shock [24,30,31,33,37,38], while eleven studies were performed on patients with established shock [10,21,23,25–27,29,30,34,35,39]. Study quality appraisal indicated that trials were of moderate quality (Table B in S2 Appendix); in particular six of them had a low risk of bias [10,22,24,27,30,32].

**Table 1:** Characteristics of included trials.

First author	Year	Setting	Study drug	Control treatment	Study drug patients	Control patients	Study drug dose	Longest follow-up	Prophylactic study drug administration?	Catecholamines before study drug administration?	Use of additional catecholamines allowed?
Albanèse J [21]	2005	Sepsis	Terlipressin	Norepinephrine	10	10	1 mg bolus, further 1 mg bolus if MAP < 65 mmHg after 20 min	Hospital stay	No	No	Yes, after the six-hours study period
Cohn SM [22]	2011	Acute traumatic injury	Vasopressin	Placebo	38	40	4 IU bolus followed by infusion of 2.4 IU/h for 5 h	30-days	No	Not specified	Not specified
Dünser MW [23]	2003	Vasodilatory shock	Vasopressin	Standard treatment	24	24	4 IU/h	ICU stay	No	Yes	Yes
Hasija S [24]	2010	Cardiac surgery	Vasopressin	Placebo	15	32	0.03 IU/min during surgery	Hospital stay	Yes	N/A	Yes
Hua F [25]	2013	Sepsis	Terlipressin	Dopamine	16	16	1.3 μg/kg/h for 48 h	28 days	No	No	Yes
Kirov MY [26]	2001	Sepsis	Methylene blue	Placebo	10	10	2 mg/kg bolus over 15 minutes, followed by 0.25–2 mg/kg/h continuous infusion for 4 hours	28 days	No	Yes	Yes
Lauzier F [27]	2006	Sepsis	Vasopressin	Norepinephrine	13	10	0.04–0.20 IU/min for 48 h	ICU stay	No	Not specified	Yes
Levin RL [28]	2004	Cardiac surgery	Methylene blue	Placebo	28	28	1.5 mg/kg/h for 1 hour	Hospital stay	No	Yes	Yes
Luckner G [29]	2006	Vasodilatory shock	Vasopressin	Standard treatment	10	8	4 IU/h	ICU stay	No	Yes	Yes
Malay MB [30]	1999	Sepsis	Vasopressin	Placebo	5	5	0.04 IU/min	24 hours	No	Yes	Yes
Maslow AD [31]	2006	Cardiac surgery	Methylene blue	Placebo	15	15	3 mg/kg bolus	Operating theatre	Yes	N/A	Yes
Memis D [32]	2002	Sepsis	Methylene blue	Placebo	15	15	0.5 mg/kg/h for 6 hours	Hospital stay		Not specified	Not specified
Morales DL [33]	2003	Cardiac surgery	Vasopressin	Placebo	17	16	0.03 IU/min for up to 72 h	Hospital stay	Yes	N/A	Yes
Morelli A DOBUPRESS [34]	2008	Sepsis	Terlipressin	Standard treatment	20	20	1 mg bolus	ICU stay	No	Yes	Yes
Morelli A TERLIVAP [35]	2009	Sepsis	Vasopressin, terlipressin	Norepinephrine	15+15	15	1.3 μg/kg/h (terlipressin), 0.03 IU/min (vasopressin) for 48 h	ICU stay	No	No	Yes
Okamoto Y [36]	2014	Cardiac surgery	Vasopressin	Placebo	49	47	1.8 U/h intraoperatively until hemodynamic stability or ICU admission	30-days	No	No	Yes
Özal E [37]	2005	Cardiac surgery	Methylene blue	Standard treatment	50	50	2 mg/kg infusion for more than 30 minutes	Hospital stay	Yes	N/A	Yes
Papadopoulos G [38]	2010	Cardiac surgery	Vasopressin	Placebo	25	25	0.03 IU/min from 30 minutes before CPB to 4 hours after CPB termination	Hospital stay	Yes	N/A	Yes
Russell JA VASST [10]	2008	Sepsis	Vasopressin	Norepinephrine	405	395	0.01–0.03 IU/min	90 days	No	Yes	Yes
Svoboda P [39]	2012	Sepsis	Terlipressin	Standard treatment	15	17	4 mg/24 h continuous infusion for 72 h	90 days	No	Yes	Yes

CPB: cardiopulmonary bypass; h: hours; ICU: intensive care unit; IU: international units; MAP: mean arterial pressure.

### Quantitative Data Synthesis

**Fig 2:**
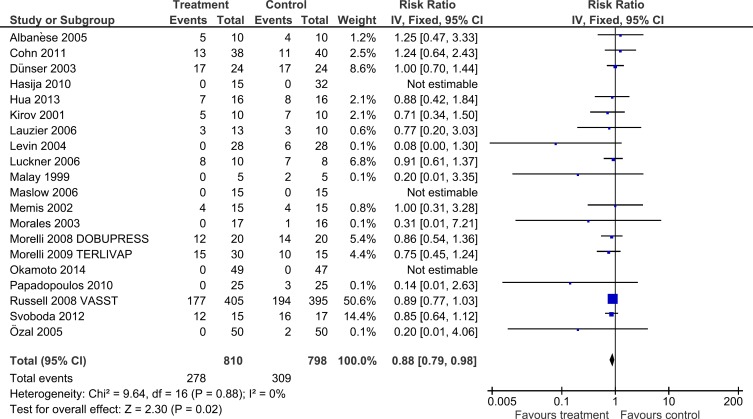
Forest plot for the risk of overall mortality.

Overall pooled analysis showed that the use of non-catecholaminergic vasopressors was associated with a significant mortality reduction (278/810 [34.3%] versus 309/798 [38.7%], RR = 0.88, 95% CI = 0.79 to 0.98, p = 0.02, I^2^ = 0%) (Table 2, Fig 2).

**Table 2:** Results of the main analysis and sub-group analyses performed.

Analysis	Number of trials	Treatment mortality	Controls mortality	RR	95% CI	P for effect	P for heterogeneity	I ^2^
**Overall**	20	34.3% (278/810)	38.7% (309/798)	0.88	0.79 to 0.98	0.02	0.88	0
**SETTING**								
**Sepsis**	10	44.5% (240/539)	51.1% (262/513)	0.87	0.77 to 0.98	0.02	0.98	0
Vasopressin / Terlipressin	8	44.9% (231/514)	51.4% (251/488)	0.87	0.77 to 0.98	0.03	0.96	0
Methylene blue	2	36% (9/25)	44% (11/25)	0.78	0.42 to 1.47	0.45	0.64	0
**Cardiac surgery**	7	0% (0/199)	5.7% (12/213)	0.16	0.04 to 0.69	0.01	0.93	0
Vasopressin / Terlipressin	4	0% (0/106)	3.4% (4/120)	0.21	0.02 to 1.74	0.15	0.72	0
Methylene blue	3	0% (0/93)	8.6% (8/93)	0.12	0.02 to 0.95	0.04	0.65	0
**DRUGS**								
**Vasopressin**	11	36.7% (226/616)	40.1% (248/617)	0.90	0.80 to 1.02	0.11	0.81	0
**Terlipressin**	5	56.7% (43/76)	66.7% (52/78)	0.85	0.69 to 1.05	0.13	0.92	0
**Vasopressin / Terlipressin**	15*	38.9% (269/692)	42.6% (290/680)	0.89	0.80 to 0.99	0.04	0.94	0
**Methylene blue**	5	7.6% (9/118)	16.1% (19/118)	0.65	0.33 to 1.29	0.22	0.34	10
**FOLLOW-UP**								
**Hospital stay**	7	5.6% (9/160)	11.4% (20/176)	0.65	0.29 to 1.47	0.30	9.30	17%
Vasopressin / Terlipressin	4	7.5% (5/67)	9.6% (8/83)	0.75	0.23 to 2.49	0.64	0.30	17%
Methylene blue	3	4.3% (4/93)	12.9% (12/93)	0.39	0.08 to 1.98	0.26	0.20	38%
**28/30-days**	6	33.6% (179/533)	37.3% (196/525)	0.88	0.76 to 1.02	0.09	0.60	0
Vasopressin / Terlipressin	5	33.3% (174/523)	36.7% (189/515)	0.88	0.76 to 1.04	0.13	0.49	0
Methylene blue	1	50% (5/10)	70% (7/10)	0.71	0.34 to 1.50	0.37	N/A	N/A
**ADDITIONAL SENSITIVITY ANALYSES**								
**Established shock**	11	46.8% (261/558)	53.2% (282/530)	0.88	0.79 to 0.98	0.02	0.98	0
Vasopressin / Terlipressin	10	46.7% (256/548)	52.9% (275/520)	0.89	0.79 to 0.99	0.03	0.98	0
Methylene blue	1	50% (5/10)	70% (7/10)	0.71	0.34 to 1.50	0.37	N/A	N/A
**Prophylactic administration**	5	0% (0/122)	4.34% (6/138)	0.20	0.04 to 1.16	0.07	0.94	0
Vasopressin / Terlipressin	3	0% (0/57)	5.5% (4/73)	0.21	0.02 to 1.74	0.15	0.72	0
Methylene blue	2	0% (0/65)	3.1% (2/65)	0.20	0.01 to 4.06	0.29	N/A	N/A
**Low risk of bias**	6	40.1% (197/491)	43% (214/497)	0.90	0.78 to 1.04	0.15	0.72	0
Vasopressin / Terlipressin	5	40.5% (193/476)	43.6% (210/482)	0.90	0.78 to 1.04	0.15	0.56	0
Methylene blue	1	26.7% (4/15)	26.7% (4/15)	1.00	0.31 to 3.28	1.00	N/A	N/A
**Versus placebo**	10	10.2% (22/217)	14.7% (34/233)	0.76	0.45 to 1.30	0.32	0.31	16
Vasopressin / Terlipressin	6	8.8% (13/149)	10.4% (17/165)	0.65	0.22 to 1.95	0.94	0.27	23
Methylene blue	4	13.2% (9/68)	25% (17/68)	0.67	0.30 to 1.52	0.34	0.26	26%
**Versus catecholamines**	5	43.7% (207/474)	49.1% (219/446)	0.88	0.77 to 1.01	0.08	0.92	0%
**Influence analysis**								
Removing *Russell JA 2008* [10]	19	25.1% (101/405)	28.7% (115/403)	0.88	0.75 to 1.02	0.09	0.84	0
Removing all other trials	All 95% CIs of RR<1 and p<0.05							

One study randomized patients to three treatment groups: terlipressin, vasopressin, and norepinephrine. CI: confidence interval; I^2^: I-squared; RR: risk ratio.

Considering the study drugs independently, all agents were associated with a non-significant trend towards improved survival of the same direction and magnitude. Indeed, vasopressin and terlipressin, considered together, were found to improve survival (269/692 [38.9%] versus 290/680 [42.6%], RR = 0.89, 95% CI = 0.80 to 0.99, p = 0.04, I^2^ = 0%, with 15 studies included) (Table 2, Figures A-D in S2 Appendix).

When analysing different settings, non-catecholaminergic vasopressors were found to reduce mortality both in sepsis (240/539 [44.5%] versus 262/513 [51.1%], RR = 0.87, 95% CI = 0.77 to 0.98, p = 0.02, I^2^ = 0%, with 10 studies included) and cardiac surgery (0/199 [0%] versus 12/213 [5.7%], RR = 0.16, 95% CI = 0.04 to 0.69, p = 0.01, I^2^ = 0%, with six studies included) (Table 2, Figures E-F in S2 Appendix). Furthermore, mortality reduction was confirmed in studies randomizing patients with established shock (261/558 [46.8%] versus 282/530 [56.2%], RR = 0.88, 95% CI = 0.79 to 0.98, p = 0.02, I^2^ = 0%, with 11 studies included) (Table 2, Figure I in S2 Appendix).

Results were not confirmed when sensitivity analyses including studies with follow-up until hospital discharge, studies follow-up until 28/30-days, studies with prophylactic administration of study drug, studies with a low risk of bias, studies with placebo as control and studies with catecholamines as control were performed (Table 2, Figures G, H, J-M in S2 Appendix). Sequential removing of each trial showed that statistical significance was lost when the study by Russell et al [10] was removed by the dataset in both the main analysis and all sub-analysis (Table 2), while removal of other trials did not alter the significance of our main analysis. The presence of a publication bias was excluded by both Begg’s and Egger’s tests (p = 0.44 and p = 0.15, respectively).

## Discussion

To the best of our knowledge, this is the largest and most comprehensive meta-analysis that investigated the effect of non-adrenergic vasopressors (i.e. vasopressin, terlipressin and methylene blue) on survival. The main finding of our study is that administration of these agents in patients with or at risk for vasodilatory shock may improve survival. The most common cause of vasodilatory shock is sepsis, whose incidence has been estimated of 751,000 cases per year in the United States and of 15,000–19,000 worldwide. [9,40,41]. However, vasodilatory shock can be related to other causes (i.e vasoplegia post-cardiotomy and exposure to cardiopulmonary bypass circuit) and be the final stage of several types of shock [9]. Despite improvement in pathophysiology understanding and therapeutic management, mortality associated with shock remains as high as 50%, and refractory cardiovascular failure is a major cause of death in the ICUs [5,42,43].

Treatment of vasodilatory shock is largely based on administration of vasopressor agents and norepinephrine is the most used. In recent years, a growing attention to the side effects of catecholamines [6] and a better understanding of the pathophysiology of shock, have led physicians to search for alternative vasopressor drugs to use in critically ill patients with severe hypotension.

At a cellular level, the most important alteration associated with vasodilatory shock is a persistent opening of ATP-sensitive K^+^-channels in the membrane of vascular smooth muscle cells. The consequent cell hyperpolarization is linked to a persistent vasodilation contributing to poor responsiveness to catecholamines in the late phase of shock [9]. Moreover there is an altered activation of inducible (NO) synthase (NOS): excess NO production leads to refractory vasodilation unresponsive to catecholamines [9]. The latter is the pathophysiological basis of persistent, refractory shock. Furthermore, following prolonged treatment with high-dose catecholamines, adrenergic receptors undergo down-regulation and desensitization [9,44].

Vasopressin, an endogenous hormone relevant for osmotic and cardiovascular homeostasis, has been extensively studied as a non-catecholamingergic vasopressor. In patients with vasodilatory shock plasma vasopressin levels are abnormally low [45], and both experimental and clinical studies have shown that vasopressin may counteract all the above described cellular mechanisms [46]. Small studies and cases series, together with the aforementioned biological reasons, pose evidence that vasopressin may be a promising agent for the management of vasodilatory shock. On the contrary, the Vasopressin in Septic Shock Trial (VASST), the largest and highest-quality RCT on this topic, failed to find any significant difference in mortality at 28- and 90-days in septic shock patients who received vasopressin or norepinephrine [10]. However, when analyzing a subgroup of patients with less severe septic shock, or receiving corticosteroid treatment, there seems to be an improved survival following vasopressin administration [10,47]. The detrimental vasoconstrictive effects of vasopressin on both heart and kidneys, is counteracted by the increased systemic mean arterial pressure, which ultimately tips the scale towards improved organ perfusion, in the context of vasodilatory shock [48,49]. Therefore, based on promising results of a recently published pilot study [50], a large multicenter randomized clinical trial has been performed [51], assessing treatment with vasopressin and corticosteroids in septic shock patients. The present study did not underline a significant survival benefit associated to vasopressin administration, however a trend towards reduced mortality was observed. Further investigations on the use of vasopressin are needed.

Terlipressin (triglycyl lysine-vasopressin) is a synthetic analogue of vasopressin with a stronger selectivity for V_1_ vasopressin receptors and a longer half-life. Currently, the main clinical indication for terlipressin administration are hepatorenal syndrome and esophageal variceal bleeding [52]. A limited number of randomized trials have investigated the role of terlipressin in the context of vasodilatory shock. Terlipressin may be more effective than vasopressin in improving hemodynamic parameters, however an excessive splanchnic vasoconstriction, decrease in cardiac output and oxygen delivery, especially following bolus injection, may be detrimental [53]. Recent trials reported that low-dose continuous infusion of terlipressin is effective in restoring blood pressure reducing adverse events [35,39]. Even though the number of studied patients is limited (153 patients randomized to terlipressin or control), the present meta-analysis confirms no increased mortality associated to terlipressin administration.

Methylene blue [12,13] restores the vascular tone by inhibiting NO synthase and soluble guanylate cyclase [55,56]. The administration of methylene blue in the treatment of several types of vasodilatory shock is exensively reported [11,13,14,56]. Nonetheless, it remains a controversial therapeutic approach with an unproven benefit [57,58].

There is general agreement that adverse effects of excessive adrenergic stimulation increases with increasing doses of catecholamines [6,7]. Therefore, survival benefit associated with non-adrenergic vasopressor use may be a consequence of their catecholamine-sparing effect, rather than a beneficial effect per se. In addition, as catecholamine are the first-line vasopressors recommended by current guidelines [3], non-adrenergic vasopressors are generally used only as rescue therapy when catecholamines alone are not sufficient. This practice is reflected in the design of the trial included in our analysis, as in only four trials patients with shock did not receive catecholamines before study drug administration [21,25,35,36], and in all trials use of catecholamines (including dobutamine) in addition to the study drugs was allowed. Ideally, an optimal trial to determine the real effect of catecholamine on mortality should compare patients receiving catecholamines with patients not receiving catecholamines at all, for example receiving levosimendan as inotropic agent to increase cardiac output instead of dobutamine or epinephrine [59,60].

Two meta-analyses on vasopressin and terlipressin use in vasodilatory shock have been recently published [54,61]. While the meta-analysis by Serpa Neto et al. showed a significant survival benefit associated with vasopressinergic agents administration, Polito et al. found no difference in mortality. Possible explanations are: inclusion of different trials, discrepancy in statistical methods, and difference in primary endpoint. Compared with these studies, our meta-analysis considers a larger and updated number of trials, not simply limited to vasopressin and terlipressin and investigates the effect of methylene blue. We acknowledge that this could lead to heterogeneity, limiting our results. We therefore performed several secondary analyses to better define the role of each agent in several setting. We found that no single agent is associated with a significant improvement of survival, although a positive trend towards mortality reduction exists for all the three drugs analysed. However, we observed a survival benefit when considering both vasopressin and terlipressin together. These results confirm the possible beneficial effect of vasopressin or terlipressin in patients with vasodilatory shock, reported in a previously published meta-analysis [54], and underlies the importance of the vasopressin system in vascular dysfunction, but also that current evidence is insufficient to recommend treatment with vasopressin or terlipressin in this category of patients.

A possible limitation of our study is the inclusion of trials performed in different clinical settings. We acknowledge that this might also be a source of heterogeneity. However, it is recognized that refractory vasodilatory shock is the final stage of shock due to any cause [8,9]. Furthermore, the pathogenesis of vasodilation in both severe sepsis and following cardiopulmonary bypass is currently considered to involve similar signaling pathways [62]. Therefore, we believe that, while keeping this limitation in mind, and in absence of more setting-specific clinical trials, our findings could be applied in every situation in which catecholamine-resistant vasodilatory shock is suspected.

Another possible limitation of our study is the inclusion of trials in which the study drug was administered before the onset of overt shock. Nevertheless, early administration of catecholamine-sparing agents has been suggested as a way to improve survival [10,51]. In addition we found that treatment with vasopressin and terlipressin was beneficial also when analyzing only patients with shock. Interestingly, we could find only one randomized trial on methylene blue in patients with shock. Another interesting point on using relatively new agents or with off label indications (as per methylene blue) is that they might be associated to unexpected side effects and complications [63].

Our study presented some additional limitations: the largest trial [10] accounts for 50% in the pooled analysis, with loss of statistical significance upon removal from the analysis; only six included trials had low bias risk and only two had randomized at least one-hundred patients. Thus, despite consistent interest in catecholamine-sparing agents, only few high-quality trials are available to provide clinicians with evidence-based indications, highlighting the need for additional large, multicenter RCTs.

## Conclusions

Our study showed that administration of non-catecholaminergic vasopressors in patients with or at risk for vasodilatory shock may improve survival. However, none of the three agents investigated, when considered alone, have been shown to reduce mortality. Furthermore, statistical significance was lost when the largest and highest-quality trial was removed from the analysis. Despite promising findings, the effects of catecholamine-sparing agents in patients with or at risk for vasodilatory shock in reducing mortality still need to be defined with proper methodology (high quality mRCTs).

## Supporting Information

S1 Appendix PRISMA Checklist. (PDF)

S2 Appendix Supplementary appendix. Supplementary information including PubMed search strategy, risk of bias assessment, supplementary tables and figures.
(DOC)
