# Supplementary material for: Non-Adrenergic Vasopressors in Patients with or at Risk for Vasodilatory Shock. A Systematic Review and Meta-Analysis of Randomized Trials
Source: PLoS One. 2015 Nov 11;10(11):e0142605. doi: 10.1371/journal.pone.0142605 (PMC4641698; doi:10.1371/journal.pone.0142605)
Supplement: S2 Appendix — Supplementary information including PubMed search strategy, risk of bias assessment, supplementary tables and figures. (DOC) [file pone.0142605.s002.doc]

**SUPPLEMENTARY APPENDIX**

**“Non-adrenergic vasopressors in patients with or at risk for vasodilatory shock. A systematic review and meta-analysis of randomized trials”.**

A. Belletti, M. Musu, S. Silvetti, O. Saleh, L. Pasin, F. Monaco, L. A. Hajjar, E. Fominskiy, G. Finco, A. Zangrillo, G. Landoni

**Search strategy for PubMed**  page 2

**Supplementary Table A** page 3

**Supplementary Table B** page 4

**Supplementary Figure A** page 10

**Supplementary Figure B** page 10

**Supplementary Figure C** page 10

**Supplementary Figure D** page 11

**Supplementary Figure E**  page 11

**Supplementary Figure F** page 12

**Supplementary Figure G** page 12

**Supplementary Figure H** page 13

**Supplementary Figure I** page 13

**Supplementary Figure J** page 14

**Supplementary Figure K** page 14

**Supplementary Figure L** page 15

**Supplementary Figure M** page 15

**Supplementary References** page 16

**Search strategy for PubMed**

(vasopressor*[ti] OR noradrenaline[ti] OR norepinephrine[ti] OR phenylephrine[ti] OR “phenylephrine hydrochloride”[ti] OR vasopressin[ti] OR “arginine vasopressin”[ti] OR glipressin[ti] OR terlipressin[ti] OR “methylene blue”[ti] OR “methylthionine chloride” [ti]) AND (randomized controlled trial [pt] OR controlled clinical trial [pt] OR randomized controlled trials [mh] OR random allocation [mh] OR double-blind method [mh] OR single-blind method [mh] OR clinical trial [pt] OR clinical trials [mh] OR (‘clinical trial’ [tw] OR ((singl* [tw] OR doubl* [tw] OR trebl* [tw] OR tripl* [tw]) AND (mask* [tw] OR blind [tw])) OR (‘latin square’ [tw]) OR placebos [mh] OR placebo* [tw] OR random* [tw] OR research design [mh:noexp] OR comparative study [mh] OR evaluation studies [mh] OR follow-up studies [mh] OR prospective studies [mh] OR cross-over studies [mh] OR control* [tw] OR prospectiv* [tw] OR volunteer* [tw]) NOT (animal [mh] NOT human [mh]) NOT (comment[pt] OR editorial[pt] OR meta-analysis[pt] OR practice-guideline[pt] OR review[pt]))

**Supplementary Table A**

List of the 23 excluded studies, together with reason for exclusion

| **First author** | **Year** | **Journal** | **Reason for exclusion** |
| --- | --- | --- | --- |
| Argenziano M [1] | 1997 | *Circulation* | No outcome data |
| Argenziano M [2] | 1998 | *J Thorac Cardiovasc Surg* | Non randomized study |
| Bennett S [3] | 2001 | *Eur J Heart Fail* | Study drug not included |
| Choong K [4] | 2009 | *Am J Respir Crit Care Med* | Pediatric population |
| Dünser MW [5] | 2004 | *Anesth Analg* | Overlapping population |
| Dünser MW [6] | 2004 | *Crit Care Med* | Overlapping population |
| Elgebaly AS [7] | 2012 | *Ann Card Anaesth* | No outcome data |
| Hajjar L [8] | 2013 | *Crit Care* | Abstract only |
| Hong SH [9] | 2012 | *J Int Med Res* | Not vasodilatory shock |
| Jeon Y [10] | 2006 | *Eur J Cardiothorac Surg* | No outcome data |
| Juffermans NP [11] | 2010 | *Nitric Oxide* | No control group |
| Koelzow H [12] | 2002 | *Anesth Analg* | Not vasodilatory shock |
| Lienhart HG [13] | 2007 | *Anaesthesist* | Study design only |
| Morelli A [14] | 2011 | *Crit Care* | No outcome data |
| Morelli A [15] | 2008 | *Crit Care* | Only catecholamines |
| Mukhtar A [16] | 2011 | *Crit Care Med* | Not vasodilatory shock |
| Mzezewa S [17] | 2004 | *Burns* | Pediatric population included |
| Park SY [18] | 2011 | *J Thorac Cardiovasc Surg* | No outcome data |
| Patel MB [19] | 2002 | *Anesthesiology* | No outcome data |
| Salman AE [20] | 2011 | *J Clin Anesth* | Non hypotensive patients |
| Van Haren RM [21] | 2013 | *J Trauma Acute Care Surg* | Preliminary results, not vasodilatory shock |
| Yildizdas D [22] | 2008 | *Intensive Care Med* | Pediatric population |
| Yimin H [23] | 2013 | *J Cardiothorac Surg* | No outcome data |

**Supplementary Table B**

Assessment of the risk of bias of included studies

| **TRIAL** | **ENTRY** | **JUDGEMENT** | **DESCRIPTION** |
| --- | --- | --- | --- |
| Albanèse J | Adequate sequence generation? | Yes | Computer generated randomization  schedule |
|  | Allocation concealment? | Yes | Allocation concealment adequate |
|  | Blinding of participants and personnel? | No | Open-label study |
|  | Blinding of outcome assessment? | No | Open-label study |
|  | Complete outcome data adressed? | Yes | No patient lost to follow-up |
|  | Free of selective reporting? | Unclear | There aren’t sufficient information to permit judgment |
|  | Free of other bias? | Yes | There is no evidence of other bias |
|  | OVERALL RISK OF BIAS | MODERATE |  |
| Cohn SM | Adequate sequence generation? | Unclear | The manuscript doesn't report the sequence generation method |
|  | Allocation concealment? | Unclear | The manuscript doesn't report allocation concealment method |
|  | Blinding of participants and personnel? | Yes | Double-blind study |
|  | Blinding of outcome assessment? | Yes | Double-blind study |
|  | Complete outcome data adressed? | Yes | No patient lost to follow-up |
|  | Free of selective reporting? | Unclear | There aren’t sufficient information to permit judgment |
|  | Free of other bias? | Yes | There is no evidence of other bias |
|  | OVERALL RISK OF BIAS | LOW |  |
| Dünser MW | Adequate sequence generation? | Unclear | The manuscript doesn't report the sequence generation method |
|  | Allocation concealment? | Unclear | The manuscript doesn't report allocation concealment method |
|  | Blinding of participants and personnel? | No | Open-label study |
|  | Blinding of outcome assessment? | No | Open-label study |
|  | Complete outcome data adressed? | Yes | No patient lost to follow-up |
|  | Free of selective reporting? | Unclear | There aren’t sufficient information to permit judgment |
|  | Free of other bias? | Yes | This study was supported in part by the Lorenz Böhler Fund |
|  | OVERALL RISK OF BIAS | MODERATE |  |
| Hasija S | Adequate sequence generation? | Yes | Computer-generated tables |
|  | Allocation concealment? | Yes | Allocation concealment adequate |
|  | Blinding of participants and personnel? | Yes | Double-blind study |
|  | Blinding of outcome assessment? | Yes | Double-blind study |
|  | Complete outcome data adressed? | Yes | No patient lost to follow-up |
|  | Free of selective reporting? | Unclear | There aren’t sufficient information to permit judgment |
|  | Free of other bias? | Yes | There is no evidence of other bias |
|  | OVERALL RISK OF BIAS | LOW |  |
| Hua F | Adequate sequence generation? | Yes | Computer-generated random number table |
|  | Allocation concealment? | Unclear | No method reported for allocation concealment |
|  | Blinding of participants and personnel? | No | The manuscript doesn’t report information about the blinding of participants and personnel. We assume this is an open-label study |
|  | Blinding of outcome assessment? | No | The manuscript doesn’t report information about the blinding of participants and personnel. We assume this is an open-label study |
|  | Complete outcome data adressed? | Yes | No patient lost to follow-up |
|  | Free of selective reporting? | Unclear | There aren’t sufficient information to permit judgment |
|  | Free of other bias? | Yes | There is no evidence of other bias |
|  | OVERALL RISK OF BIAS | MODERATE |  |
| Kirov MY | Adequate sequence generation? | Unclear | The manuscript doesn't report the sequence generation method |
|  | Allocation concealment? | Yes | Unmarked, sealed envelopes |
|  | Blinding of participants and personnel? | No | Open-label study |
|  | Blinding of outcome assessment? | No | Open-label study |
|  | Complete outcome data adressed? | Yes | No patient lost to follow-up |
|  | Free of selective reporting? | Unclear | There aren’t sufficient information to permit judgment |
|  | Free of other bias? | Yes | There is no evidence of other bias |
|  | OVERALL RISK OF BIAS | MODERATE |  |
| Lauzier F | Adequate sequence generation? | Yes | Quote: "Computer-generated block randomization list for each center was prepared by a pharmacist not involved in patient recruitment” |
|  | Allocation concealment? | Yes | Quote: “Randomization was concealed using numbered, opaque sealed envelopes” |
|  | Blinding of participants and personnel? | No | Open-label study |
|  | Blinding of outcome assessment? | No | Open-label study |
|  | Complete outcome data adressed? | Yes | Analysis by ITT. One patient allocated to norepinephrine was excluded because next of kin did not provide consent |
|  | Free of selective reporting? | Unclear | There aren’t sufficient information to permit judgment |
|  | Free of other bias? | Yes | There is no evidence of other bias |
|  | OVERALL RISK OF BIAS | LOW |  |
| Levin RL | Adequate sequence generation? | No | Methods used for randomization was the hospital admission number |
|  | Allocation concealment? | Unclear | No method reported for allocation concealment |
|  | Blinding of participants and personnel? | No | The manuscript doesn’t report information about the blinding of participants and personnel. We assume this is an open-label study |
|  | Blinding of outcome assessment? | No | The manuscript doesn’t report information about the blinding of participants and personnel. We assume this is an open-label study |
|  | Complete outcome data adressed? | Yes | No patient lost to follow-up |
|  | Free of selective reporting? | Unclear | There aren’t sufficient information to permit judgment |
|  | Free of other bias? | Yes | There is no evidence of other bias |
|  | OVERALL RISK OF BIAS | HIGH |  |
| Luckner G | Adequate sequence generation? | Yes | Random number generating computer program |
|  | Allocation concealment? | Unclear | No method reported for allocation concealment |
|  | Blinding of participants and personnel? | No | Open-label study |
|  | Blinding of outcome assessment? | No | Open-label study |
|  | Complete outcome data adressed? | No | Quote: “Cardiovascular function could not be stabilized adequately by incremental dosages of norepinephrine in one patient randomly assigned to the norepinephrine group, but MAP could be restored with supplementary AVP infusion. This patient was therefore switched to the AVP/norepinephrine group for statistical evaluation” |
|  | Free of selective reporting? | Unclear | There aren’t sufficient information to permit judgment |
|  | Free of other bias? | Yes | There is no evidence of other bias |
|  | OVERALL RISK OF BIAS | MODERATE |  |
| Malay MB | Adequate sequence generation? | Yes | Computer-generated list |
|  | Allocation concealment? | Unclear | No method reported for allocation concealment |
|  | Blinding of participants and personnel? | Yes | Double-blind study |
|  | Blinding of outcome assessment? | Yes | Double-blind study |
|  | Complete outcome data adressed? | Yes | No patient lost to follow-up |
|  | Free of selective reporting? | Unclear | There aren’t sufficient information to permit judgment |
|  | Free of other bias? | Yes | There is no evidence of other bias |
|  | OVERALL RISK OF BIAS | LOW |  |
| Maslow AD | Adequate sequence generation? | Unclear | The manuscript doesn't report the sequence generation method |
|  | Allocation concealment? | Unclear | No method reported for allocation concealment |
|  | Blinding of participants and personnel? | No | Open-label study |
|  | Blinding of outcome assessment? | No | Open-label study |
|  | Complete outcome data adressed? | Yes | No patient lost to follow-up |
|  | Free of selective reporting? | Unclear | There aren’t sufficient information to permit judgment |
|  | Free of other bias? | Yes | There is no evidence of other bias |
|  | OVERALL RISK OF BIAS | MODERATE |  |
| Memis D | Adequate sequence generation? | Yes | Computer-steered permuted block design |
|  | Allocation concealment? | Unclear | No method reported for allocation concealment |
|  | Blinding of participants and personnel? | Yes | Double-blind study |
|  | Blinding of outcome assessment? | Yes | Double-blind study |
|  | Complete outcome data adressed? | Yes | No patient lost to follow-up |
|  | Free of selective reporting? | Unclear | There aren’t sufficient information to  permit judgment |
|  | Free of other bias? | Yes | There is no evidence of other bias |
|  | OVERALL RISK OF BIAS | LOW |  |
| Morales DL | Adequate sequence generation? | Unclear | Quote: "Using the method of simple random sampling” |
|  | Allocation concealment? | Unclear | No method reported for allocation concealment |
|  | Blinding of participants and personnel? | Yes | Double-blind study |
|  | Blinding of outcome assessment? | Yes | Double-blind study |
|  | Complete outcome data adressed? | No | Quote: “Two patients in the placebo group could not be weaned from CPB because of intractable vasodilatory shock and received vasopressin, which is the standard of care at our institution. Additionally, 4 patients from the vasopressin group were disqualified when the study drug was inadvertently discontinued during the first 6 hours, out of protocol. The responses of the remaining 27 patients were analyzed” |
|  | Free of selective reporting? | Unclear | There aren’t sufficient information to permit judgment |
|  | Free of other bias? | Yes | There is no evidence of other bias |
|  | OVERALL RISK OF BIAS | MODERATE |  |
| Morelli A DOBUPRESS | Adequate sequence generation? | Yes | Computer-based procedure |
|  | Allocation concealment? | Unclear | No method reported for allocation concealment |
|  | Blinding of participants and personnel? | No | Open-label study |
|  | Blinding of outcome assessment? | No | Open-label study |
|  | Complete outcome data adressed? | No | One patient excluded from the analysis |
|  | Free of selective reporting? | Unclear | There aren’t sufficient information to permit judgment |
|  | Free of other bias? | Yes | There is no evidence of other bias |
|  | OVERALL RISK OF BIAS | MODERATE |  |
| Morelli A TERLIVAP | Adequate sequence generation? | Yes | Computer-based procedure |
|  | Allocation concealment? | Unclear | No method reported for allocation concealment |
|  | Blinding of participants and personnel? | No | The manuscript doesn’t report information about the blinding of participants and personnel. We assume this is an open-label study |
|  | Blinding of outcome assessment? | No | The manuscript doesn’t report information about the blinding of outcome assessment. We assume this is an open-label study |
|  | Complete outcome data adressed? | Unclear | No patient lost to follow-up |
|  | Free of selective reporting? | Unclear | There aren’t sufficient information to permit judgment |
|  | Free of other bias? | Yes | There is no evidence of other bias |
|  | OVERALL RISK OF BIAS | MODERATE |  |
| Okamoto Y | Adequate sequence generation? | Yes | Computer-generated randomization list |
|  | Allocation concealment? | No | Quote: “One researcher who was not involved in the surgery made up the drug protocol according to the computer-generated randomization list for each patient” |
|  | Blinding of participants and personnel? | Yes | Double-blind study |
|  | Blinding of outcome assessment? | Yes | Double-blind study |
|  | Complete outcome data adressed? | No | 8 patients excluded from the analysis following randomization (3 withdrew consent) |
|  | Free of selective reporting? | Unclear | There aren’t sufficient information to permit judgment |
|  | Free of other bias? | Yes | There is no evidence of other bias |
|  | OVERALL RISK OF BIAS | MODERATE |  |
| Özal E | Adequate sequence generation? | Yes | Table of random digits |
|  | Allocation concealment? | Unclear | No method reported for allocation concealment |
|  | Blinding of participants and personnel? | No | The manuscript doesn’t report information about the blinding of participants and personnel. We assume this is an open-label study |
|  | Blinding of outcome assessment? | No | The manuscript doesn’t report information about the blinding of participants and personnel. We assume this is an open-label study |
|  | Complete outcome data adressed? | Yes | No patient lost to follow-up |
|  | Free of selective reporting? | Unclear | There aren’t sufficient information to permit judgment |
|  | Free of other bias? | Yes | There is no evidence of other bias |
|  | OVERALL RISK OF BIAS | MODERATE |  |
| Papadopoulos G | Adequate sequence generation? | Unclear | The manuscript doesn't report the sequence generation method |
|  | Allocation concealment? | Unclear | No method reported for allocation concealment |
|  | Blinding of participants and personnel? | Yes | Double-blind study |
|  | Blinding of outcome assessment? | Yes | Double-blind study |
|  | Complete outcome data adressed? | Yes | No patient lost to follow-up |
|  | Free of selective reporting? | Unclear | There aren’t sufficient information to permit judgment |
|  | Free of other bias? | Yes | There is no evidence of other bias |
|  | OVERALL RISK OF BIAS | MODERATE |  |
| Russell JA VASST | Adequate sequence generation? | Yes | A computer-generated randomization list of variable permuted blocks of 2, 4, and 6 was used for treatment allocation |
|  | Allocation concealment? | Yes | Central telephone randomization system |
|  | Blinding of participants and personnel? | Yes | Double-blind study |
|  | Blinding of outcome assessment? | Yes | Double-blind study |
|  | Complete outcome data adressed? | Yes | Analysis by ITT. One patient lost to follow-up; two withdrew consent. Exclusion of these patients is unlikely to have influenced results |
|  | Free of selective reporting? | Yes |  |
|  | Free of other bias? | Yes | There is no evidence of other bias |
|  | OVERALL RISK OF BIAS | LOW |  |
| Svoboda P | Adequate sequence generation? | Yes | Computer-generated list |
|  | Allocation concealment? | Yes | Opaque, sealed, envelopes |
|  | Blinding of participants and personnel? | No | The manuscript doesn’t report information about the blinding of participants and personnel. We assume this is an open-label study |
|  | Blinding of outcome assessment? | No | The manuscript doesn’t report information about the blinding of participants and personnel. We assume this is an open-label study |
|  | Complete outcome data adressed? | Yes | Two patients were excluded from analysis; however mortality outcome for these two patients has been reported |
|  | Free of selective reporting? | Unclear | There aren’t sufficient information to permit judgment |
|  | Free of other bias? | No | The study was supported by a grant of IGA MZ CR NR 9284-3 |
|  | OVERALL RISK OF BIAS | MODERATE |  |

**Supplementary Figure A –** Forest plot for mortality - Vasopressin

**Supplementary Figure B –** Forest plot for mortality - Terlipressin

**Supplementary Figure C –** Forest plot for mortality - Vasopressin + terlipressin

**Supplementary Figure D –** Forest plot for mortality - Methylene blue

**Supplementary Figure E –** Forest plot for mortality – Sepsis

**Supplementary Figure F –** Forest plot for mortality – Cardiac surgery

**Supplementary Figure G –** Forest plot for mortality - Follow-up: hospital stay

**Supplementary Figure H** – Forest plot for mortality - Follow-up: 28/30 days

**Supplementary Figure I** – Forest plot for mortality - Patients with established shock

**Supplementary Figure J** – Forest plot for mortality - Prophylactic drug administration

**Supplementary Figure K** – Forest plot for mortality - Studies with low risk of bias

**Supplementary Figure L** – Forest plot for mortality - Studies with placebo as control

**Supplementary Figure M** – Forest plot for mortality - Studies with catecholamine as control

**Supplementary References**

1. Argenziano M, Choudhri AF, Oz MC, Rose EA, Smith CR, Landry DW. A prospective randomized trial of arginine vasopressin in the treatment of vasodilatory shock after left ventricular assist device placement. Circulation. 1997;96(9 Suppl):II-286-90.

2. Argenziano M, Chen JM, Choudhri AF, Cullinane S, Garfein E, Weinberg AD, et al. Management of vasodilatory shock after cardiac surgery: identification of predisposing factors and use of a novel pressor agent. J Thorac Cardiovasc Surg. 1998;116:973-80.

3. Bennett S, McKeown J, Drew P, Griffin S. Angiotensin in cardiac surgery: efficacy in patients on angiotensin converting enzyme inhibitors. Eur J Heart Fail. 2001;3:587-92.

4. Choong K, Bohn D, Fraser DD, Gaboury I, Hutchison JS, Joffe AR, et al. Vasopressin in pediatric vasodilatory shock: a multicenter randomized controlled trial. Am J Respir Crit Care Med. 2009;180:632-9.

5. Dünser MW, Fries DR, Schobersberger W, Ulmer H, Wenzel V, Friesenecker B, et al. Does arginine vasopressin influence the coagulation system in advanced vasodilatory shock with severe multiorgan dysfunction syndrome? Anesth Analg. 2004;99:201-6.

6. Dünser MW, Hasibeder WR, Wenzel V, Schwarz S, Ulmer H, Knotzer H, et al. Endocrinologic response to vasopressin infusion in advanced vasodilatory shock. Crit Care Med. 2004;32:1266-71.

7. Elgebaly AS, Sabry M. Infusion of low-dose vasopressin improves left ventricular function during separation from cardiopulmonary bypass: a double-blind randomized study. Ann Card Anaesth. 2012;15:128-33.

8. Hong SH, Lee JM, Choi JH, Chung HS, Park JH, Park CS. Perioperative assessment of terlipressin infusion during living donor liver transplantation. J Int Med Res. 2012;40:225-36.

9. Jeon Y, Ryu JH, Lim YJ, Kim CS, Bahk JH, Yoon SZ, et al. Comparative hemodynamic effects of vasopressin and norepinephrine after milrinone-induced hypotension in off-pump coronary artery bypass surgical patients. Eur J Cardiothorac Surg. 2006;29:952-6.

10. Juffermans NP, Vervloet MG, Daemen-Gubbels CR, Binnekade JM, de Jong M, Groeneveld AB. A dose-finding study of methylene blue to inhibit nitric oxide actions in the hemodynamics of human septic shock. Nitric Oxide. 2010;22:275-80.

11. Koelzow H, Gedney JA, Baumann J, Snook NJ, Bellamy MC. The effect of methylene blue on the hemodynamic changes during ischemia reperfusion injury in orthotopic liver transplantation. Anesth Analg. 2002;94:824-9, table of contents.

12. Hajjar L, Vincent JL, Rhodes A, Annane D, Galas F, Almeida J, et al. Vasopressin Versus Norepinephrine for the Management of Shock After Cardiac Surgery (VaNCS study): a randomized controlled trial. Crit Care. 2013;17(Suppl 2):P222.

13. Lienhart HG, Wenzel V, Braun J, Dörges V, Dünser M, Gries A, et al. [Vasopressin for therapy of persistent traumatic hemorrhagic shock: The VITRIS.at study]. Anaesthesist. 2007;56:145-8, 150.

14. Morelli A, Donati A, Ertmer C, Rehberg S, Kampmeier T, Orecchioni A, et al. Effects of vasopressinergic receptor agonists on sublingual microcirculation in norepinephrine-dependent septic shock. Crit Care. 2011;15:R217.

15. Morelli A, Ertmer C, Rehberg S, Lange M, Orecchioni A, Laderchi A, et al. Phenylephrine versus norepinephrine for initial hemodynamic support of patients with septic shock: a randomized, controlled trial. Crit Care. 2008;12:R143.

16. Mukhtar A, Salah M, Aboulfetouh F, Obayah G, Samy M, Hassanien A, et al. The use of terlipressin during living donor liver transplantation: Effects on systemic and splanchnic hemodynamics and renal function. Crit Care Med. 2011;39:1329-34.

17. Mzezewa S, Jönsson K, Aberg M, Sjöberg T, Salemark L. A prospective double blind randomized study comparing the need for blood transfusion with terlipressin or a placebo during early excision and grafting of burns. Burns. 2004;30:236-40.

18. Park SY, Kim DH, Kim JS, Lim SH, Hong YW. Comparative effects of norepinephrine and vasopressin on internal thoracic arterial graft flow after off-pump coronary artery bypass grafting. J Thorac Cardiovasc Surg. 2011;141:151-4.

19. Patel BM, Chittock DR, Russell JA, Walley KR. Beneficial effects of short-term vasopressin infusion during severe septic shock. Anesthesiology. 2002;96:576-82.

20. Salman AE, Salman MA, Saricaoglu F, Akinci SB, Aypar Ü. Pain on injection of propofol: a comparison of methylene blue and lidocaine. J Clin Anesth. 2011;23:270-4.

21. Van Haren RM, Thorson CM, Ogilvie MP, Valle EJ, Guarch GA, Jouria JA, et al. Vasopressin for cerebral perfusion pressure management in patients with severe traumatic brain injury: preliminary results of a randomized controlled trial. J Trauma Acute Care Surg. 2013;75:1024-30; discussion 1030.

22. Yildizdas D, Yapicioglu H, Celik U, Sertdemir Y, Alhan E. Terlipressin as a rescue therapy for catecholamine-resistant septic shock in children. Intensive Care Med. 2008;34:511-7.

23. Yimin H, Xiaoyu L, Yuping H, Weiyan L, Ning L. The effect of vasopressin on the hemodynamics in CABG patients. J Cardiothorac Surg. 2013;8:49.
